# P45 Lupus flare with myositis: an atypical presentation in the absence of elevated creatine kinase

**DOI:** 10.1093/rap/rkae117.076

**Published:** 2024-11-05

**Authors:** Shumaila Baloch, Siwalik Banerjee

**Affiliations:** Warwick Hospital, Warwick, United Kingdom; University Hospital Coventry and Warwickshire, Coventry, United Kingdom

## Abstract

**Introduction:**

Systemic lupus erythematosus (SLE) is a multi-systemic disease, but the coexistence of inflammatory myositis in SLE has not been extensively studied. Myositis, a rare complication, is typically associated with elevated creatine kinase (CK) levels. We report a case of SLE presenting with a lupus flare and an atypical manifestation of myositis, confirmed by MRI despite low CK levels. This case underscores the complex nature of lupus flares and the challenges in diagnosing and managing such atypical presentations. The diagnostic dilemma of myositis with low CK levels adds a layer of complexity to understanding and treating SLE-related complication.

**Case description:**

A 25-year-old lady, known patient of systemic lupus erythematosus (SLE), positive ANA, dsDNA, and anti-Ro antibodies, managed with hydroxychloroquine, experienced generalized body stiffness, aches, and lower limb weakness for the past 3 months. She was admitted with worsening lower limb pain and weakness, oral ulcers, arthralgias, myalgias and alopecia despite intramuscular steroids.

On examination, her lower limb power was reduced to 3/5 with normal sensation. A detailed neurological assessment was difficult due to severe pain. Her chest was clear with normal heart sounds, and the rest of the examination was unremarkable.

Her erythrocyte sedimentation rate (ESR) was 69 mm/hr, and her C-reactive protein (CRP) was 50 mg/L, with a normal white cell count and normal kidney and liver function. Her urine protein-creatinine ratio (PCR) was 50.9 mg/mmol. An ECG and chest X-ray were unremarkable.

Initially, she was treated for a urinary tract infection (UTI) due to urinary symptoms, and further investigations were done to check for myositis and a lupus flare. Her CK was low at 36 U/L, with normal lactate dehydrogenase (LDH). Her complement levels were low with raised anti-dsDNA levels.

An MRI of the spine was normal. Given the history and clinical signs suggestive of myositis, an MRI of the hip was performed, revealing active myositis.

She was started on IV methylprednisolone for the active flare. A renal opinion was sought due to the positive urine PCR, and they advised a possible biopsy. However, her repeat urine PCR came back negative, and the renal team concluded that the abnormal result was likely secondary to the UTI, negating the need for a biopsy.

Her condition and muscle power improved (4+/5) with steroid and mycophenolate mofetil. Upon tapering the steroid to 10 mg, her arthralgia recurred, but myositis was improving. Further follow-up has been arranged.

**Discussion:**

This case highlights the complex nature of systemic lupus erythematosus (SLE) and the challenges in managing its multi-systemic manifestations. The patient’s presentation with generalized body stiffness, lower limb weakness, and pain, alongside oral ulcers, arthralgias, myalgias, and alopecia, exemplifies the diverse clinical spectrum of SLE.

The initial differential diagnosis included a lupus flare and myositis. The elevated ESR and CRP levels, alongside low complement levels and increased anti-dsDNA titres, were indicative of active disease. Interestingly, the patient’s CK levels were low, which devi-ates from the typical presentation of myositis. Moreover, specific myositis antibodies were absent. Despite low CK, the clinical suspicion of myositis prompted an MRI scan of thigs, which confirmed active inflammation in the hip muscles.

Another important aspect of this case was the initial positive urine PCR, which raised concerns about renal involvement—a serious complication of SLE. However, the resolution of this abnormality upon treating a concurrent UTI highlights the importance of re-evaluating initial findings and considering alternative explanations before proceeding with invasive procedures such as a renal biopsy.

In summary, this case tells us the importance of a comprehensive and dynamic approach in the management of SLE. It illustrates the need for continuous monitoring, the utility of advanced imaging techniques in diagnosing myositis, and the cautious interpretation of laboratory results in the context of coexisting conditions. The patient’s favorable response to treatment also emphasizes the efficacy of current therapeutic strategies in controlling disease activity and preventing long-term complications

**Key learning points:**

• Lupus flares can present with a wide range of symptoms, including musculoskeletal manifestations such as myositis.

• The absence of typical markers, such as elevated CK and specific antibodies, should not rule out the possibility of myositis in lupus patients.

• Prompt recognition and management of myositis in lupus flares are crucial to prevent further complications and improve patient outcomes.

• Effective management of SLE often requires collaboration among various specialists. In this case, the involvement of the renal team was crucial for further evaluation and management, demonstrating the value of a coordinated care approach.

In conclusion, this case highlights the complexity of lupus flares and the importance of considering myositis as a potential complication, even in the absence of typical markers. Early recognition and appropriate management are key to improving outcomes for patients with lupus-associated myositis.

